# Association of Paracentral Acute Middle Maculopathy with Visual Prognosis in Retinal Artery Occlusion: A Retrospective Cohort Study

**DOI:** 10.1155/2022/9404973

**Published:** 2022-05-21

**Authors:** Siying Liang, Qingshan Chen, Chenli Hu, Miaohong Chen

**Affiliations:** Vitreoretinal Department, Shenzhen Eye Hospital, Shenzhen Eye Institute, Jinan University, Shenzhen 518040, China

## Abstract

**Background:**

The association between paracentral acute middle maculopathy (PAMM) and visual acuity in patients with retinal artery occlusion (RAO) is unknown. This study explored the clinical features and prognostic factors for visual acuity of RAO accompanied by PAMM.

**Methods:**

This retrospective study included patients with RAO who underwent FFA and OCT examinations at Shenzhen Eye Hospital from July 2015 to June 2019. The changes in vision and macular structure were observed.

**Results:**

Eighty-eight patients were included. There were 58 patients (65.9%) with central RAO (CRAO) and 30 (34.1%) with branch RAO (BRAO). Fifty-two eyes were diagnosed with PAMM, of which 33 eyes (63.5%) were from CRAO patients and 19 (36.5%) were from BRAO patients. At diagnosis, the PAMM group had significantly better logMAR BCVA values than the no-PAMM group (median (IQR), 1.35 (0.725–2) vs. 2.15 (1.47–2.3), *P*=0.002). In addition, the PAMM group had significantly better logMAR BCVA values during follow-up than the no-PAMM group (median (IQR), 1 (0.05–2) vs. 2 (1.15–2.3), *P*=0.001). After adjustment for age, gender, CRAO/BRAO, comorbidities, and symptom duration, PAMM was associated with good visual acuity improvement (RR = 3.29, 95% CI: 1.29–8.37, *P*=0.013).

**Conclusion:**

PAMM was associated with good visual acuity improvement during follow-up in patients with RAO.

## 1. Introduction

Retinal artery occlusion (RAO) leads to a blockage of the arterial blood supply to the retina, leading to retinal ischemia or infarction and transient or permanent vision loss [1–3]. RAO is analogous to a cerebral stroke localized to the eye [1, 2]. The incidence is 1/100,000 [1]; it can occur at any age but is more common in older people [2]. The occlusion can occur within the central retinal artery (CRAO), a branch retinal arteriole (BRAO), or the cilioretinal artery. An embolus from carotid artery stenosis or a cardioembolic source is the most common cause of RAO [1, 2]. Giant cell arteritis, although an uncommon cause, should always be considered. Less common causes include thrombophilia, vasospasm, and other vasculitic or inflammatory disorders [1–3]. It manifests as a sudden, painless, monocular vision loss or degradation [1, 2]. The diagnosis is based on funduscopic findings and fundus fluorescein angiography (FFA) [1–3]. Optical coherence tomography (OCT) can also help diagnose eye pathologies [4–6]. Management includes ocular massage, isovolumic hemodilution, carbogen inhalation or hyperbaric oxygen, drugs, paracentesis, and embolectomy, but their effectiveness is limited [1, 2, 7–13]. CRAO results in visual acuity of 20/400 or worse in 80% of the cases, while BRAO results in 20/40 or worse in 90% [1, 2].

Paracentral acute middle maculopathy (PAMM) is found by OCT in patients with retinal capillary ischemia [14]. PAMM can be an isolated phenomenon or a complication of a retinal vasculopathy or systemic condition [14–18]. Funduscopic examinations are usually negative because PAMM manifests on OCT as subtle parafoveal lesions deep within the retina [14, 16, 17]. Therefore, its management usually involves the treatment of the underlying condition. Partial resolution is the most usual outcome, and significant visual impairment is uncommon [14, 17].

The association between PAMM and visual acuity in patients with RAO is unknown. Arteritic CRAO carries the worst prognosis [19], while spontaneous reperfusion, presence of a cilioretinal artery (found in about 50% of the individuals), and shorter duration of occlusion are known to improve prognosis [1, 2]. Still, the predictors or risk factors for eye vision improvement in patients with RAO are poorly known. Pielen et al. [20] showed that chronic heart disease (CHD), occlusion-to-treatment time, and smoking were associated with prognosis. Yilmaz et al. [21] showed that the baseline foveal disorganization of retinal inner layers (DRIL) score assessed using OCT had some prognostic values in patients with CRAO.

Therefore, this study aimed to explore the clinical features and prognostic factors for visual acuity of RAO accompanied by PAMM. The results could help determine the patients' prognosis and adopt a management strategy.

## 2. Materials and Methods

### 2.1. Study Design and Population

This retrospective study included patients with RAO [1–3] who underwent FFA and OCT examinations at the Ophthalmology Department of Shenzhen Eye Hospital from July 2015 to June 2019. The inclusion criteria were acute vision loss or visual field defect within 2 weeks and FFA showing prolonged retinal artery filling time. The patients with any other ocular diseases that affected vision were excluded. This study was approved by the Medical Ethics Committee of Shenzhen Eye Hospital Affiliated to Jinan University. The requirement for patients' informed consent was waived due to the retrospective nature of the study.

### 2.2. Data Collection

Age, gender, and comorbidities might influence the visual outcomes and fundus appearance. In addition, RAO ultimately affects the visual acuity and changes in the OCT macular structure of the patient. Therefore, these variables were analyzed in this study. Data including age, gender, comorbidities, vision, best-corrected visual acuity (BCVA), treatment strategies, and the changes in the macular structure assessed by OCT were collected from the medical charts.

Two doctors were presented with OCT and OCTA explorations of the patients and asked to evaluate them for the presence of PAAM; if OCT showed high reflection in the retinal core layer, PAMM was diagnosed; if OCT showed full-layer retinal edema with blurred layers, PAMM was ruled out. Cohen's kappa coefficient was used to determine consistency. The results showed that the two physicians considered 50 subjects to have PAMM and 33 patients to be without. Cohen's Kappa coefficient of the two doctors was 0.882 (95% CI: 0.782–0.981, *P* < 0.001), showing strong consistency (Table 1). When the judgment of the two doctors was inconsistent, senior doctors were asked to evaluate and classify the inconsistent eyes. Hence, the inconsistent five eyes were reevaluated by doctor 3, of which two were judged to be PAMM and three were judged to be no-PAMM.

### 2.3. Statistical Analysis

Statistical analysis was performed using SPSS 18.0 (IBM, Armonk, NY, USA). Continuous data with a normal distribution were presented as means ± standard deviation and analyzed using the independent sample *t*-test; otherwise, they were presented as median (IQR) and analyzed using the Mann–Whitney *U* test. Categorical data were presented as *n* (%) and analyzed using the chi-square test. Multivariable logistic regression analysis was performed for the adjustment of potential confounders (i.e., age, gender, CRAO/BRAO, comorbidities, and duration of symptoms). Two-sided *P* values <0.05 were considered statistically significant.

## 3. Results

During the study period, 122 eligible patients underwent FFA and OCT examinations and were diagnosed with RAO. Thirty-four patients were excluded: 16 patients were lost to follow-up (13 were not willing to come back to the hospital for examination, and 3 patients were treated in other hospitals), 10 patients had a macular disease (macular degeneration and hiatus), 4 patients had other fundus lesions (retinal vein occlusion and arteritis), and 4 patients had glaucoma, had undergone glaucoma surgery, and received long-term glaucoma drugs. Therefore, 88 patients were finally included. There were no significant differences in age, gender, duration of symptoms, and comorbidities between the PAMM and no-PAMM groups (all *P* > 0.05) (Table 2).

**Figure 1:**
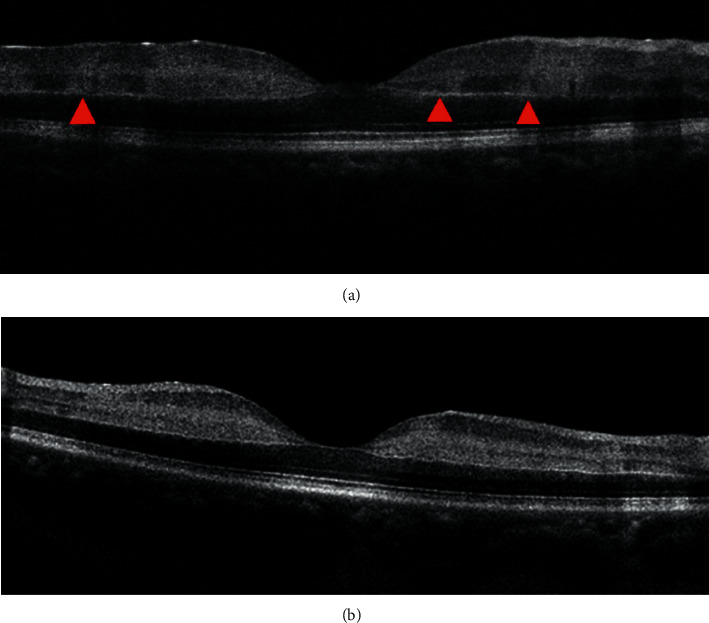
Paracentral acute middle maculopathy (PAMM) manifestation by optical coherence tomography (OCT) examination. (a) Mild edema between the retinal layers, with multiple side-by-side strong reflection bands in the inner nuclear retinal layer (INL) near the center of the macula, extending upward to involve the ganglion cell layer (red triangle). (b) Both INL and inner plexiform layer (IPL) reflections are enhanced.

**Figure 2:**
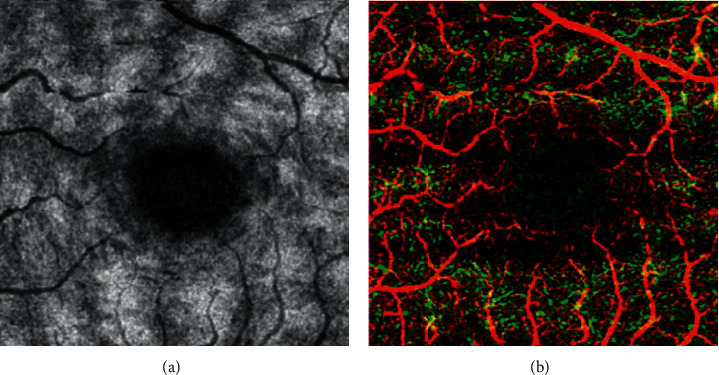
Paracentral acute middle maculopathy (PAMM) manifestation by optical coherence tomography angiography (OCTA) examination. (a) Enface image of the middle layer of the retina, with multiple patchy strong reflection lesions in the center of the macula. (b) Deep capillary plexus (DCP) blood flow density in the macular area decreases and the foveal avascular zone (FAZ) area increases.

There were 58 patients (65.9%) with CRAO and 30 (34.1%) with BRAO. Fifty-two eyes were diagnosed with PAMM, of which 33 eyes (63.5%) were from CRAO patients and 19 (36.5%) were from BRAO patients. Figures 1 and 2 show the typical changes in PAMM. At diagnosis, the PAMM group had significant better logMAR BCVA values than the no-PAMM group (median (IQR), 1.35 (0.725–2) vs. 2.15 (1.47–2.3), *P*=0.002) (Table 2). This study used the Snellen visual acuity meter to test visual acuity and performed logMAR conversion. Finger count was recorded as 20/3200 and hand movement and light perception as 20/4000. The PAMM group had better logMAR BCVA values during follow-up than the no-PAMM group (median (IQR), 1 (0.05–2) vs. 2 (1.15–2.3), *P*=0.001) (Table 3). After adjustment for age, gender, CRAO/BRAO, comorbidities, and symptom duration, PAMM was associated with good visual acuity improvement (RR = 3.29, 95% CI: 1.29–8.37, *P*=0.013).

## 4. Discussion

This study aimed to explore the clinical features and prognostic factors for visual acuity of RAO accompanied by PAMM. The results suggest that PAMM is associated with good visual acuity improvement during follow-up in patients with RAO.

Previous studies of the prognostic factors of RAO suggested that arteritic RAO has the worst prognosis among all types of RAO [19, 22]. In such patients, anti-inflammatory drugs should be started as soon as possible to reduce arterial inflammation [23]. The cilioretinal artery is present in about 50% of the population [24, 25]. In patients with RAO, the cilioretinal artery, if present, can provide blood to the retina, resulting in less severe ischemia and better outcomes [1, 2]. A shorter interval between symptom onset and treatment was associated with a better visual prognosis [1, 2, 20]. Smoking and CHD have been associated with poor outcomes, and these two factors are well-known to be associated with the outcomes of ischemic diseases [20]. None of these factors were associated with logMAR BCVA during follow-up in the present study. A study showed that irreversible retinal damage appeared at 4 h after occlusion [26]. The median time from symptom to treatment was 29 h, indicating that most patients had irreversible retinal damage, probably reducing the impact of other factors.

PAMM is associated with various conditions like retinal vein occlusion, diabetic retinopathy, BRAO or CRAO, sickle cell retinopathy, migraine, oral contraceptives, and various medical procedures [15, 27–29]. A series reported that the most common cause of PAMM was BRAO [30]. In the present study, the presence of PAMM was associated with good visual acuity at diagnosis and good visual outcomes during follow-up. Some of the DRIL score features overlap the diagnostic criteria for PAMM [31–33]. These studies showed that the DRIL score was associated with the visual outcomes after RAO [21]. The DRIL has a prognostic value in various eye diseases [31–37]. Ahn et al. [38] showed that the final central macular thickness was associated with a very poor visual prognosis in RAO, as supported by Chen et al. [39]. In this study, PAMM might be due to hypoperfusion and hypoxia, and the blood vessels are not completely blocked, leading to hypoxia. Timely treatment in the hypoxia state leads to relatively good visual recovery. In patients without PAMM, full-thickness retinal edema and complete lack of oxygen (anoxia) to the cells might result in poorer visual recovery. Although RAO has a poor visual prognosis, this study suggests that the presence of PAAM in the eyes with RAO is associated with a better chance of visual improvement. Therefore, patients with PAAM in the setting of a RAO should be treated more actively. Therefore, patients with PAMM and RAO can be treated more actively, including with the use of intravenous thrombolytic therapy, oxygen inhalation, vasodilation, and intraocular pressure reduction. For patients undergoing thrombolysis, attention should be paid to the patient's coagulation parameters, whether there is a bleeding tendency, and whether the liver and kidney functions are affected. But this study did not carry out a detailed analysis of the standard of treatment, which is a limitation.

This study has limitations. The study was retrospective, limiting the data to those found in the charts and routinely assessed. It was a single-center study with a small sample size. No biomarkers were examined. Although diabetic retinopathy is associated with RAO and PAMM, it was not specifically examined in the present study because it was not systematically investigated in the included patients. A future prospective study should include DR and functional examinations such as electrophysiological tests. Prospective multicenter randomized clinical trials with a large sample size are needed to provide high-level evidence.

## 5. Conclusions

This study suggests that PAMM is associated with good visual acuity improvement during follow-up in patients with RAO. These results suggest that patients with RAO and PAMM might benefit from more aggressive management. Still, interventions need to be examined in future studies.

## Figures and Tables

**Table 1:** Consistency between the two doctors for the evaluation of PAMM.

		Doctor 1	
		PAMM	No-PAMM
Doctor 2	PAMM	50	1
	No-PAMM	4	33

**Table 2:** Demographic and clinical characteristics of patients with retinal artery occlusion.

Clinical features	All (*n* = 88)	PAMM (*n* = 52)	No-PAMM (*n* = 36)	*P*
Age, year, mean ± SD	55.7 ± 16.4	53.7 ± 17.5	58.6 ± 14.5	0.174^a^
Gender, male, *n* (%)	6 2 (70%)	38 (73%)	24 (66.7%)	0.510^b^
Duration of symptoms (h), median (IQR)	29 (17–144)	26 (11–168)	32 (24–120)	0.915^c^
Comorbidities, *n* (%)	51 (58%)	28 (53.8%)	23 (63.9%)	0.348^b^
LogMAR best-corrected visual acuity, range		0–2.5	0.1–2.5	
LogMAR best-corrected visual acuity, median (IQR)		1.35 (0.725–2)	2.15 (1.47–2.3)	0.002^c^

^a^Independent sample *t*-test; ^b^chi-square test; ^c^Mann–Whitney *U* test. PAMM, paracentral acute middle maculopathy; IQR, interquartile range; BCVA, best-corrected visual acuity.

**Table 3:** Differences in clinical features during follow-up before discharge between groups with and without PAMM clinical data in patients with retinal artery occlusion.

Clinical features	PAMM	No-PAMM	*P*
Vision examination			
LogMAR best-corrected visual acuity (range)	0–2.3	0.05–2.5	
LogMAR best-corrected visual acuity, median (IQR)	1 (0.05–2)	2 (1.15–2.3)	<0.001^c^
Diagnosis, *n* (%)	52 (59%)	36 (41%)	
CRAO	33	25	
BRAO	19	11	

PAMM, paracentral acute middle maculopathy; BCVA, best-corrected visual acuity; IQR, interquartile range; OCT, optical coherence tomography; CRAO, central retinal artery occlusion; BRAO, branch retinal artery occlusion.

## Data Availability

The datasets used and/or analyzed during the current study are available from the corresponding author upon request.

## Abbreviations

PAMM: Paracentral acute middle maculopathy
RAO: Retinal artery occlusion
CRAO: Central RAO
BRAO: Branch RAO
FFA: Fundus fluorescein angiography
OCT: Optical coherence tomography
CHD: Chronic heart disease
DRIL: Disorganization of retinal inner layers
BCVA: Best-corrected visual acuity.
